# Current practices of partner notification among MSM with HIV, gonorrhoea and syphilis in the Netherlands: an urgent need for improvement

**DOI:** 10.1186/1471-2334-12-114

**Published:** 2012-05-14

**Authors:** Fleur van Aar, Imke Schreuder, Yolanda van Weert, Ralph Spijker, Hannelore Götz, Eline Op de Coul

**Affiliations:** 1 Epidemiology & Surveillance department, Centre for Infectious Disease Control, National Institute of Public Health and the Environment, Bilthoven, the Netherlands; 2 Department of Virology, Erasmus MC, Rotterdam, the Netherlands; 3 STI AIDS Netherlands, Amsterdam, the Netherlands; 4 Department of Infectious Disease Control – division STI/HIV, GGD Rotterdam-Rijnmond, Municipal Public Health Service Rotterdam-Rijnmond, Rotterdam, the Netherlands

**Keywords:** Partner notification, MSM, HIV, Syphilis, Gonorrhoea, The Netherlands

## Abstract

**Background:**

Partner notification (PN) among individuals newly diagnosed with HIV/STI is seen as a vital tool to identify others at risk of infection. However, hardly any data are available on the effectiveness of PN on HIV/STI transmission in the Netherlands. This study aims to fill this gap by assessing current PN practices, case-finding effectiveness, and determinants of being notified among men having sex with men (MSM) in the Netherlands.

**Methods:**

Nurses from five STI centers participated in a prospective pilot study on PN outcomes (partners being: at risk, notifiable, notified, and tested) for HIV/STI, by completing a newly developed PN registration form (PN database). PN outcomes including case-finding effectiveness (number of newly diagnosed cases in partners/number of partners being tested) for HIV, syphilis, and gonorrhoea were studied among MSM. Furthermore, the national STI database was analyzed to identify determinants of being notified. The number of infections that remained undetected was estimated based on these two databases.

**Results:**

In total 105 MSM, newly diagnosed with HIV/STI, reported 612 sexual partners at risk of whom 41% were notifiable and 31% were notified. Patient referral was the predominant PN method (90%). The overall case-finding percentage was 36% (HIV: 15-33%, gonorrhoea: 17-50% and syphilis: 4-11%). Case-finding percentages were lower in the national STI database: 21% (5%, 28%, 12%). Persons with one or more sexual partners, known HIV positives, and IDU were more likely to be notified to the STI clinic. Notified clients were more likely to have HIV/STI than unnotified clients (OR 1.7-2.5). Based on these two databases, an estimated 75 to 133 infections remained undetected (HIV: 12–90; gonorrhoea: 28–97; syphilis: 5–12 infections).

**Conclusions:**

Partner notification among MSM in the Netherlands is suboptimal; an extensive number of STI/HIV infections remained undetected mainly due to unnotifiable partners. To enhance PN practices, combined and innovative PN interventions such as Internet-based PN will be implemented for hard-to-reach MSM and other risk groups.

## Background

The increasing trends of sexually transmitted infections (STI) in the Netherlands since the mid 1990s have been explained as a result of an increased risk behavior, which has been associated with human immunodeficiency virus (HIV) treatment optimism and an improved quality of life after the introduction of combination antiretroviral therapy (cART) [1]. Also, a more active testing policy has contributed to increasing numbers of HIV cases and other STI [2,3]. Since 2005, the annual number of newly diagnosed HIV infections has been more than 1,100 per year and is particularly high among men having sex with men (MSM, 68% of all new diagnoses in 2010) [2,3]. Today, MSM account for 89% of all syphilis cases and 57% of gonorrhoea in the Netherlands [3].

An estimated 8,000 to 10,000 people - 40% of all people living with HIV in the Netherlands - are unaware of their infection [4]. Previous studies showed that people who are unaware of HIV and those with a primary infection may contribute up to 90% of the new HIV infections [2,5,6]. Furthermore, almost 40% of HIV infected MSM in the Netherlands were diagnosed in a late stage of infection [2]. It is essential, for individuals and public health that HIV infections are detected early, preferably during the primary phase when people are most infectious and likely unaware of the risk they pose to others. Moreover, a number of STI may facilitate the spread of HIV and their control could also reduce HIV transmission [7].

Partner notification (PN) can be a vital tool to control transmission of HIV/STI. PN is a process in which sexual partners of newly diagnosed individuals (index patients) are informed of their exposure to an infection and need to visit a health service. PN may increase the proportion of cases being aware of the potential risk of infection. This subsequently increases the proportion of cases seeking care which can reduce transmission of HIV/STI on public health level [8]. Several studies have shown that PN among index patients is effective as a case-finding tool in HIV/STI prevention [9-17].

Four methods to notify sexual partners have been distinguished: (1) provider referral in which sexual partners are notified through care providers (2) patient referral in which the index is responsible for notifying sexual partners (3) contract referral where sexual partners are initially notified by the index, but the health professional takes over if the index fails to notify partners by the predetermined date and (4) network notification in which (anonymous) sexual partners are notified at location or within sexual networks (e.g. saunas, darkrooms) [18].

Studies have shown that provider referral is more effective than patient referral in terms of numbers of partners notified and presenting for testing [10,13,19-22]. Nevertheless, patient referral is more commonly performed in the Netherlands, due to the labour intensity of PN and other barriers that PN poses to health care providers [11,18,21]. Partner notification for STI/HIV in the Netherlands is conducted by health care professionals at STI centres, general medical practices, hospitals and HIV treatment centres. General practitioners (GPs) fulfil an important role in STI care, complementary to STI centres. It has been estimated that GPs are responsible for 70% of STI-related episodes and 80-85% of STI diagnoses [23]. For HIV, it has been estimated that ± 28% of all new HIV cases are diagnosed by GPs, 25% in hospitals and 22% at STI centres. Remaining HIV cases are diagnosed at other locations or by pregnancy screening [24]. Details on PN methods are described by STI in the national guideline for partner notification for STI/HIV [18].

In national expert meetings organised by the National Institute for Public Health and the Environment (RIVM) and the Erasmus Medical Center, it was acknowledged that studies assessing PN effectiveness in the Netherlands are lacking and that an evaluation of current PN approaches is needed [25]. In order to develop new PN interventions, it is essential to understand the determinants that affect PN outcomes.

Recently, a Dutch PN group was formed including professionals from five STI centres, the RIVM and STI AIDS Netherlands, to start a three-year pilot study on current and enhanced PN practices and outcomes. Here, we describe the results of the first year of data collection among MSM to provide insight in the case-finding effectiveness of current PN practices. Furthermore, data from the national STI surveillance system were analysed to explore case-finding among notified clients on national scale and to identify determinants of being notified.

## Methods

To assess PN outcomes (see § ‘PN registration form’) of current PN practices in the Netherlands, we analysed data from two databases:

1) *PN database* based on a newly developed registration form implemented in STI centres in five pilot regions. Using this database, PN outcomes and case-finding effectiveness for HIV, syphilis, and gonorrhoea were studied among MSM. Case-finding effectiveness was defined as the number of newly diagnosed cases in partners divided by the number of partners being tested.

2) *National STI database* including data on STI testing among notified and unnotified clients from all 26 STI centers [3]. The national STI database was analyzed to identify determinants of being notified.

Furthermore, the number of infections that remained undetected was estimated based on the two databases by comparing the same five regions.

### Data collection

#### PN database

Data collection started in 2010 in the STI centers of The Hague, Rotterdam, Arnhem, Brabant and Groningen to evaluate outcomes of current PN practices (the ‘baseline’ situation: before implementation of enhanced PN strategies) [22] and future enhanced PN strategies (Internet-based PN and PN training for STI nurses).

A uniform registration form was developed and piloted by the PN group to monitor PN outcomes in terms of the numbers of eligible partners (partners ‘at risk’), notifiable partners (partners are reachable through an address, email or phone number), notified partners and tested partners. The first part of the form includes information on methods of notification (e.g. patient or provider referral), demographic characteristics of index cases (such as gender, age, risk group), and numbers and types (casual/regular, anonymous) of partners. For each notifiable partner, the second part of the form was completed for the partners’ characteristics (such as gender, meeting location, type of sex contact) and STI testing (test result and location of test). This information was reported by the index case during the consultation in which PN was being addressed. The partner information was linked to the index case by a unique client identifier.

One supervisor per STI centre, also member of the PN group, was appointed to implement the registration form among all nurses conducting PN. Data collection was conducted by these nurses from index patients at the first or follow-up visits (or in case there was no follow-up visit: by telephone). Data collection started with newly diagnosed HIV as a priority disease, followed by syphilis and gonorrhoea cases. MSM of 16 years or older were eligible as index patient. Due to large numbers, forms were not completed for chlamydia unless co-diagnosed with HIV, syphilis or gonorrhoea. Partners at risk were defined as partners with whom the index had unprotected anal or oral intercourse during the last 4 weeks up to 1 year, depending on the type of STI [18]. Data on testing results was mainly collected during follow-up visits (in case partners joint the consultation) or by telephone after the notification.

#### National STI database

To explore determinants of being notified and case-finding effectiveness among notified and unnotified MSM on national level, the STI surveillance database was analysed (2008–2010) [3]. The database includes sociodemographic characteristics such as gender, ethnicity (country of birth of client and both parents), age, socioeconomic status (SES, based on average income per household, paid job and education level, http://www.scp.nl), sexual preference, and commercial sex work, sexual risk (condom use with casual and steady partner), and STI diagnoses (HIV, gonorrhoea, syphilis, chlamydia, and hepatitis B virus (HBV)). The variable ‘notified yes/no’ in this database registers PN either by another health care professional or an index patient. It refers to clients being notified for an STI test and can distinguish notified clients from clients who visit the centre on their own initiative.

### Data analysis

Numbers of notified sexual partners and case-finding effectiveness from the PN database were studied by descriptive statistics including characteristics of index patients and partners. We estimated the numbers of detected infections and infections that remained undetected due to partners being unnotifiable. The number of infections for all partners at risk was estimated, assuming that the proportion of infections among unnotified partners was similar to notified partners for whom a test result was available. Corrections were made for unknown test results and the proportion of notified MSM who decided not to seek STI testing. These estimates were based on different case finding percentages (using different denominators). The first included notified partners who had a test result for the particular STI that was diagnosed among the index patient. However, partners who were notified for a particular STI may have been at risk for other STI. For that reason STI clinics test all MSM for all main STI (including HIV). Therefore, we also calculated case finding percentages based on all notified partners with an STI test result (irrespective of the STI that they were at risk for/notified for). Finally, case finding percentages of the PN registration were compared with the case finding percentages in the national STI database.

To study determinants of being notified, MSM aged 16–70 years who were tested for STI/HIV were selected in the national STI database. Multivariate logistic regression analyses were conducted to assess the associations between the outcomes HIV, gonorrhoea, syphilis, chlamydia and HBV with being ‘notified yes/no’. The crude associations from univariate analyses were corrected for possible confounders: age, socio-economic status (SES), ethnicity, history or symptoms of STI(s), previously HIV diagnosed, commercial sex worker (CSW), client of CSW, condom use with last sexual partner, and number of partners in past six months. Inclusion of variables was repeated until the regression coefficient changed less than 10%. Secondly, multivariate logistic regression analyses using backward selection were conducted to investigate associations between being notified and socio-demographic and behavioural variables. All analyses were performed in SPSS 18.

## Results

### PN database

#### Characteristics of index patients

Between January 2010 and March 2011, 105 registration forms were collected from MSM index patients. The STI centers of The Hague, Rotterdam, Arnhem, Brabant and Groningen returned respectively 31, 21, 10, 17 and 21 registration forms (5 from other centers). Of these index patients, 18% (n = 19) were younger than 25 years old; the majority (50%) was between 26 and 45 years old. Most MSM (84%) were national born and 6% had a Surinamese or Antillean background (10% other).

For 23 index patients, PN could not be conducted due to refusal to cooperate (n = 8), being non-reachable (n = 7), and other reasons not to participate (n = 8).

Information on numbers and types of partners was available for 91% of the index MSM (96/105). Forty-five percent of the MSM had a regular partner and one or more casual partner(s), 41% had only casual partner(s), and 11% had only a regular partner.

A total of 69 MSM were HIV infected of whom 84% were newly diagnosed. Of these, 70% had co-infections with gonorrhoea (n = 20), syphilis (n = 20) or chlamydia (n = 18) (Table 1). Sixteen MSM were diagnosed with only gonorrhoea, 13 with syphilis only, and seven had multiple STI.

**Table 1:** STI/HIV infections among MSM index patients (n = 105)

	***N***	***%***
**Total index population**	105	100
**Total HIV + infections**	69	65.7 (100)
*Known HIV+*	11	16.0
*Newly diagnosed HIV+*	58	84.0 (100)
*Recent HIV infection*	19	32.8
**Total gonorrhoea infections**	42	40.0
**Total syphilis infections**	36	34.3
**Only one infection**		
*HIV*	21	20.0
*Gonorrhoea*	16	15.2
*Syphilis*	13	12.4
**Co infection**	55	52.4 (100)
*HIV positive*	48	87.0 (100)
+ Gonorrhoea	20	41.7
+ Syphilis	20	41.7
+ Chlamydia	18	37.5
*HIV negative*	7	13.0

Of all index patients, 23% was notified themselves. Of these, 11 were newly HIV diagnosed; one was recently infected (< 6 months). Of these eleven HIV + MSM, 6 were not notified for HIV but for another STI.

#### PN outcomes

**Figure 1:**
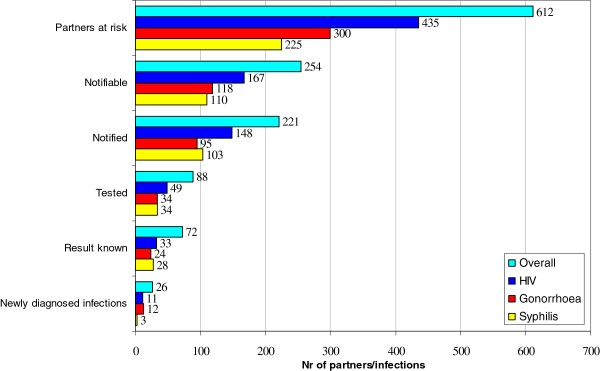
Case-finding effectiveness of PN among MSM.

Index patients (n = 96) reported a total of 612 sexual partners at risk for HIV/STI of whom 254 (41%) were notifiable and 221 were notified (36%) (Figure 1). These findings imply that 64% (n = 391) of the partners at risk were lost along the PN process, mainly due to anonymity (being ‘unnotifiable’). However, when partners were notifiable it was likely that they were notified (87%). For 86 index patients, detailed information on type of partners was available (Table 2). Of these, a total of 210 partners was reported of which 79% was casual. Of these, 36% were met through friends, 30% were contacted through Internet and 22% were gained in bars/clubs, (sex) parties or saunas. Index patients reported that for 70% of the casual partners the last sexual contact was unprotected.

**Table 2:** Partner notification outcomes among notifiable partners (n = 210)

	**N (%)**	***N (%)Regular partners***	***N (%)Casual partners***	***p-value***
**Total population of notifiable partners**	210 (100)	39 (18.6)	165 (78.6)	
**Safe sex (last intercourse)**	208 (99.0*)			
Protected	22 (10.5)	9 (23.1)	13 (7.9)	
Unprotected	147 (70.0)	27 (69.2)	115 (69.7)	<0.03
Unknown	39 (18.6)	3 (7.7)	36 (21.8)	
**Partners notified**	210 (100)			
Yes	193 (91.9)	34 (87.2)	154 (93.3)	ns
No	17 (8.1)	5 (12.8)	11 (6.7)	
**Method of PN**	193 (91.9*)			
Patient referral	174 (90.2)	29 (85.3)	140 (90.9)	
Provider referral	16 (8.3)	4 (11.8)	12 (7.8)	ns
Other	3 (1.5)	1 (2.9)	2 (1.3)	
**Means of PN**	193 (100)			
Face-to-face	82 (42.5)	32 (94.1)	47 (30.5)	
Telephone	67 (34.7)	2 (5.9)	64 (41.6)	<0.001
Internet	34 (17.6)	0 (0.0)	33 (21.4)	
Unknown	10 (5.2)	na	na	
**Partner tested**	193 (100)			
Yes	88 (45.6)	28 (71.8)	57 (34.5)	
No	13 (6.7)	5 (12.8)	9 (5.5)	na
Unknown	92 (47.7)	6 (15.4)	89 (53.9)	
**Partner tested at**	88 (100)			
STI clinic	41 (46.6)	25 (64.1)	16 (9.7)	
HIV treatment clinic	3 (3.4)	1 (2.6)	2 (1.2)	<0.01
General Practitioner	11 (12.5)	1 (2.6)	9 (5.5)	
Other/Unknown	33 (37.5)	1 (2.6)	31 (18.8)	

na: not available; ns: not significant; * outcomes not 100% due to missing data.

Ninety percent of the sexual partners were notified through patient referral (Table 2). Most commonly used PN methods were face-to-face (42%) or telephone conversations (35%, including short text messages). Internet was often used to notify casual partners (21%).

For 46% of the notified partners it was known if they were examined for an STI. Since STI diagnoses among partners were mostly reported by the index (in some cases confirmed by the STI clinic) test results were more often available for regular partners compared to casual partners.

Test percentages where higher for those referred by the provider (62%, n = 10) compared to those referred by the index (44%, n = 76). The majority of the partners was tested at an STI clinic (47%). Six percent did not seek STI testing after being notified.

#### Case-finding effectiveness

Among the 72 partners for whom test results were available, 26 infections were identified (11 HIV, 12 gonorrhoea and 3 syphilis, Table 3). The 11 HIV infected partners were notified by six index patients who were all newly HIV diagnosed and 4 of them had a co-infection. One index with a recent HIV infection had four partners who became newly diagnosed with HIV.

**Table 3:** Case-finding effectiveness and estimated (un)detected infections*

	**PN registration**							**National STI database**		
	**CF (%)**		**Detected Infections (N)**	Estimated*detected infections*for all notified partners* (N)		Maximum*undetectedinfections*for all non-notified partners* (N)		**CF (%)**	Estimated*detected infections*for all notified partners* (N)	Minimum*undetected infections*for all non-notified partners* (N)
	**Estimate 1**^**a**^	**Estimate 2**^**b**^		**Estimate 1**^**a**^	**Estimate 2**^**b**^	**Estimate 1**^**a**^	**Estimate 2**^**b**^			
Overall	36.1	36.1	26	75	75	**133**	**133**	20.6	43	**75**
HIV	15.3	33.3	11	21	46	**41**	**90**	4.6	6	**12**
GO	16.7	50.0	12	15	45	**32**	**97**	14.7	13	**28**
SYPH	4.2	10.7	3	4	10	**5**	**12**	4.0	4	**5**

*corrected for 6% who decided not to seek STI testing, and chlamydia and HBV infections excluded; CF: case-finding effectiveness.

a) denominator: all notified partners with an STI test result, irrespective of the STI that they were at risk for/notified for.

b) denominator : notified partners who had a test result for the particular STI that was diagnosed among the index patient.

The overall case-finding effectiveness among partners who were tested after being notified was 36%. For 6% of the partners it was known that they did not seek STI testing. The number of *detected* infections among all notified partners was estimated at 75–133, if test results would have been available for all of them (Table 3). Furthermore, the number of undetected infections was estimated for all unnotified partners (including notifiable partners that were not notified and unnotifiable – often anonymous - partners); assuming the same case-finding effectiveness as among partners who were notified and tested. The maximum number of expected infections among all 612 partners at risk was 208; meaning that 133 infections would be additionally detected if all partners at risk were notified and tested. Similarly, 41–90 HIV-, 32–97 gonorrhoea- and 5–12 syphilis infections may have remained undetected. These findings were based on two calculations on case-finding percentages in the PN database. The percentages were lower in the national STI database (Table 4). Therefore, the same calculation was repeated using the percentages from the national STI database. Comparing these estimates, the maximum range of undetected infections was for HIV: 12–90; gonorrhoea: 28–97; and syphilis: 5–12 (Table 3).

**Table 4:** Number of MSM being tested for STI/HIV in the national STI database and case-finding effectiveness

	***N included***	***Case finding effectiveness (%)***	***Logistic regression***		
			**Multivariate**		
			**OR (95%CI)**		**p-value**
**HIV**	38,879	2.4			
Unnotified	33,924	2.1	1.0		
Notified	4,955	**4.6**	2.3* (1.9-2.6)		<0.001
**Chlamydia**	48,657	10.6			
Unnotified	42,019	9.6	1.0		
Notified	6,638	**16.8**	2.0** (1.8-2.1)		<0.001
**Gonorrhoea**	48,649	8.4			
Unnotified	42,012	7.4	1.0		
Notified	6,637	**14.7**	2.5*** (2.3-2.7)		<0.001
**Syphilis**	48,532	3.0			
Unnotified	41,929	2.8	1.0		
Notified	6,603	**4.0**	1.7*** (1.5-2.0)		<0.001
**Hepatitis B**	20,726	0.8			
Unnotified	17,679	0.7	1.0		<0.001
Notified	3,047	**1.2**	1.7* (1.2-2.5)		

* No adjustments; ** Adjusted for previous STI, Known HIV + and symptoms;

*** Adjusted for symptoms; CI: confidence interval; OR: odds ratio.

### National STI database

#### Case-finding effectiveness

On national scale, 49,675 MSM attended an STI center of who 28%, 33% and 39% visited these centers in 2008, 2009 and 2010 respectively. Of these MSM, 13% were notified due to a potential STI risk. Forty-five percent visited the center in Amsterdam, after which The Hague (7%) and Rotterdam (5%) were the most visited centers by MSM.

In total, 10,022 MSM were diagnosed with at least one STI (20%). Of all MSM, 7,659 were previously diagnosed with HIV and 941 were newly HIV diagnosed. Furthermore, 4,111 gonorrhoea-, 1,439 infectious syphilis-, 5,204 chlamydia-, and 157 HBV- infections were reported.

Multivariate analyses indicated that clients who have been notified compared to unnotified clients were more likely to have an HIV positive test result (OR 2.3 [95% CI 1.9-2.6]) and a positive HBV test result (OR 1.7 [1.2-2.5], Table 4); while no confounding was found. The ORs for a chlamydia, gonorrhoea and syphilis infection were after adjusting for confounding: 2.0 [1.8-2.1], 2.5 [2.3-2.7], and 1.7 [2.3-2.7], respectively.

#### Determinants of being notified

Characteristics differed significantly between clients being notified for an STI and clients who visited the center on their own initiative (Table 5). Persons who have had one or more sexual partner(s) from a high-risk group (OR: 1.4 [1.2-1.5]), those with a known HIV positive status (OR: 1.4 [1.3-1.5]), and clients who injected drugs in the past six months (OR 2.6 [1.5-4.4]) were more likely of being notified compared to persons without these characteristics. Persons who didn’t have any sexual partners in past six months (OR: 0.5 [0.3-0.7]), clients who had clinical symptoms of STI (OR: 0.6 [0.6-0.6]), and CSW and clients of CSW were less likely to be notified (both ORs: 0.5 [0.4-0.6]).

**Table 5:** Determinants of being notified for MSM visiting STI centers, 2008-2010

**Variable**	**N**	**%**	**OR (95%CI)**	**p-value**
**Total population**	48,956	100		
**Age group**				
16-25	8,153	16.7	1.0	-
26–35	13,436	27.4	**1.1** (1.0-1.2)	0.03
36–45	14,676	30.0	**1.1** (1.0-1.2)	0.02
46+	12,437	25.4	0.9 (0.9-1.0)	0.12
**Known HIV positive**				
No	40,895	83.5	1.0	-
Yes	7,649	15.6	**1.4** (1.3-1.5)	<0.001
**Previous STI (< 2 years)**				
No	36,866	75.3	1.0	-
Yes	10,784	22.0	**1.1** (1.0-1.2)	0.01
**Symptoms**				
No	35,001	71.5	1.0	-
Yes	13,519	27.6	**0.6** (0.6-0.6)	<0.001
Partner from risk group*				
No	5,499	11.2	1.0	-
Yes	42,879	87.6	**1.4 (**1.2-1.5)	0.001
**Number of partners (<6 months)**				
1	4,565	9.3	1.0	-
0	376	0.8	**0.5** (0.3-0.7)	<0.001
2–5	15,168	31.0	1.0 (0.9-1.1)	0.62
6–10	6,609	13.5	0.9 (0.8-1.0)	0.13
>10	16,706	34.1	0.9 (0.8-1.0)	0.11
**Condom Use (last intercourse)**				
No	10,475	21.4	1.0	-
Yes	8,130	16.6	**0.8** (0.8-0.9)	<0.001
**IDU**				
No	48,245	98.5	1.0	-
Yes, ever	158	0.3	**1.6** (1.0-2.3)	0.03
Yes < past 6 months	69	0.1	**2.6** (1.6-4.5)	<0.001
**CSW**				
No	47,932	97.9	1.0	-
Yes	883	1.8	**0.5** (0.4-0.6)	<0.001
**Client of CSW**				
No	47,855	97.8	1.0	-
Yes	955	2.0	**0.5** (0.4-0.6)	<0.001
**Year of consultation**				
2008	13,698	28.0	1.0	-
2009	16,133	33.0	**0.9** (0.8-1.0)	<0.01
2010	19,125	39.1	**0.9** (0.9-1.0)	0.03
**Region**				
North. Holland + Flevo	24,360	49.8	1.0	-
South. Holland	7,393	15.1	0.9 (0.8-1.0)	0.23
East. Netherlands	6,324	12.9	1.0 (0.9-1.1)	0.77
Zeeland + Brabant	4,125	8.4	1.0 (0.9-1.1)	0.57
Utrecht	2,794	5.7	**0.7** (0.6-0.8)	<0.01
Limburg	2,085	4.3	**0.7** (0.6-0.9)	<0.01
North. Netherlands	1,815	3.7	**0.7** (0.6-0.8)	<0.01

High risk group: MSM (men having sex with men), IDU (injecting drug user), CSW (commercial sex worker), person origination from endemic country. Non significant variables not shown (anonymous, swinger, SES, ethnicity). CI: confidence interval.

## Discussion

This study is one of the first describing PN practices and the main case-finding effectiveness among MSM in the Netherlands. A large gap (58%) was demonstrated between the numbers of sexual risk partners and notifiable partners (42%). Of the notifiable partners, 87% was notified. Although the case finding percentage was high in the PN registration (36%), we estimated that a higher number of infections remained undiagnosed in the 5 pilot regions.

The large gap between the numbers of partners at risk and notifiable partners is to great extent due to anonymous sexual partners. The results demonstrate an urgent need for the development of innovative PN methods to reach anonymous, possibly high-risk, partners. By the end of 2011, an Internet-based PN web application will be implemented as a pilot project in Rotterdam and Amsterdam to help to bridge this gap. The application provides the opportunity to send messages through email, SMS, chat boxes and/or dating sites, which is expected to be accepted by the target group as has been described in literature [26].

The difference between notifiable and notified partners is considerably smaller than the difference between partners at risk and notifiable partners. Furthermore, patient referral was performed in 90% of all notified partners. These results suggest that MSM index patients are willing to notify their partners given that they are notifiable. From literature, however, it appeared that provider referral is the most effective method in terms of numbers of partners notified and tested [10,13,17-22]. We also showed that testing rates among partners were significantly higher when they were notified by a professional (63% vs. 44%). Provider referral might be an effective method when index patients do not have the intention or ability to notify partners. Also, provider referral has the benefit that an immediate appointment for STI testing can be made with notified partner(s). Health care professionals indicated that provider referral is at times desirable but patient referral is often used due to lack of time and high work load. The willingness of index patients to self-notify sexual partners is in line with other publications [27]. Another study showed that 77% of the index patients rated patient referral as a good method of PN while only 6% was negative about PN [28], which underlines a potential success of Internet-based PN by the index patient.

The HIV-, gonorrhoea- and syphilis case-finding percentages of respectively 15-33%, 17-50% and 4-11% indicate that PN was successful in detecting new infections. However, case-finding percentages from the national STI database were considerably lower, resulting in wide ranges of estimated (un)detected infections. Also in other studies case-finding percentages varied widely across STI and studies. By example, Brewer [12] showed that percentages of initiated contacts newly diagnosed for syphilis, gonorrhoea and HIV were respectively 8% (range 1-23%), 18% (range 8-34%) and 8% (0.2-48%). Various methods of measuring PN case-finding effectiveness were reported, which hampers the comparison of our study with other studies.

The eleven newly diagnosed HIV infections among partners were related to six index patients, who were all newly diagnosed with HIV and four were co-infected with other STI. No HIV diagnoses were found among partners of index patients with a known HIV infection and co-infected with an STI. It is unknown whether this is due to a small risk due to cART of the index patient or the fact that partners of known HIV-infected MSM who are co-infected with and STI are not always notified for the HIV-infection. We assume that the HIV case-finding effectiveness of PN might be lower for index cases with a known HIV infection compared to newly HIV diagnosed index patients. More information on recent and longstanding HIV infections among index patients should be collected in the near future to indicate priority cases and to improve PN services. It could be considered to offer provider referral to HIV cases to ensure notification of (casual) partners and to attain the highest possible test percentages among partners. Within the framework of limited time and high work load of professionals in care, individuals with a recent HIV infection and newly diagnosed in general should have priority over the known HIV positives. Knowing that in the Netherlands high-risk core groups contribute to HIV transmission among MSM [29], the results also underline the importance of (additional) sexual network contact tracing.

Our study results should be interpreted in the context of some limitations. Between 2008 and 2010, a majority of MSM visited the STI center in Amsterdam, although these data were not available in the PN database due to another system of registration. Since only 5 out of 26 centers participated in the pilot and the number of new diagnoses is only a small part of all STI diagnoses made nationwide; data are likely not representative for the whole country. Numbers of index MSM with HIV are fairly complete for the 5 participating STI centers, since the numbers in the PN registration form were similar to numbers in the national STI database. Syphilis and gonorrhoea cases included in the analyses were less complete. In 2010, 268 gonorrhoea- and 89 infectious syphilis infections were reported among MSM in the five centers as registered in the national database.

Furthermore, we cannot exclude that PN results were subjected to information or recall bias. Index patients might report (un)intentionally less sexual partners at risk, or might report more partners notified and tested than actually are. For about 60% of the partners it was unknown whether they sought HIV/STI testing. Index patients might have selected partners who were most at risk or the reported partners’ test results might be from more ‘close’ contacts who may have been more (recently) exposed, resulting in a higher case-finding percentage. Hence, the assumption of the same case-finding percentages among all partners - as used in the estimates - might be inaccurate. Conversely, the majority of unnotifiable partners were most likely anonymous, among whom HIV/STI incidences might be higher [15]. Also, the case-finding effectiveness per STI might be higher if casual partners were also exposed to an STI by other sexual partners.

In conclusion, our results underline the need to improve PN practices in the Netherlands to reduce the number of unnotifiable partners and to increase numbers of notified and tested partners. The impact of PN on the prevention of STI/HIV transmission is likely to be small when PN coverage is low. Partners who are not notifiable pose a challenge to PN, but the plans to provide PN through the Internet could further reduce the number of unidentified infections. In addition, STI AIDS Netherlands, the RIVM and the PN working group are currently developing other methods to enhance PN in the Netherlands. Starting in October 2011, all nurses from the 5 participating STI centers will be offered a newly developed training that will focus on improvement of time management during the STI consultation and the reduction of barriers for PN. This training will be offered as a follow-up course on the motivational interviewing course which has been offered to all STI nurses in the Netherlands. Furthermore, PN practices have been improved already simply by the implementation of the PN registration form, as reported by the PN working group. Next year outcomes of PN practices will be evaluated again after the implementation of these new methods. Further studies and PN enhancements should also focus on PN outcomes of general practitioners (GPs) and collaboration between disciplines (HIV treatment centers, STI centers and GPs).

## Competing interests

The authors declare that they have no competing interests.

## Authors’ contributions

FA, IS and EC analysed and interpreted the data and drafted the manuscript. Other authors were involved in the data acquisition and contributed to drafting and revision of the paper. All authors read and approved the final manuscript.

## ‡ Partner Notification Group

A. Casanovas (Public Health Service (PHS), Amsterdam), M. Hulstein (PHS: ‘Veiligheids - en Gezondheidsregio’ Gelderland Midden (VGGM), D. van Veldhuizen (VGGM), J. Rodriquez (PHS, The Hague), L. Vasen (PHS Rotterdam-Rijnmond), Y. van Weert (RIVM, Bilthoven), E. Op de Coul (RIVM, Bilthoven), R. Spijker (STI AIDS Netherlands, Amsterdam), F. van Aar (RIVM, Bilthoven)

## Pre-publication history

The pre-publication history for this paper can be accessed here:

http://www.biomedcentral.com/1471-2334/12/114/prepub
